# Association between green tea and coffee consumption and body iron storage in Japanese men and women: a cross-sectional study from the J-MICC Study Saga

**DOI:** 10.3389/fnut.2023.1249702

**Published:** 2023-08-10

**Authors:** Hinako Nanri, Megumi Hara, Yuichiro Nishida, Chisato Shimanoe, Chiharu Iwasaka, Yasuki Higaki, Keitaro Tanaka

**Affiliations:** ^1^Department of Physical Activity Research, National Institutes of Biomedical Innovation, Health, and Nutrition, Osaka, Japan; ^2^Laboratory of Gut Microbiome for Health, Microbial Research Center for Health and Medicine, National Institutes of Biomedical Innovation, Health, and Nutrition, Osaka, Japan; ^3^Department of Preventive Medicine, Faculty of Medicine, Saga University, Saga, Japan; ^4^Department of Pharmacy, Saga University Hospital, Saga, Japan; ^5^Laboratory of Exercise Physiology, Faculty of Sports and Health Science, Fukuoka University, Fukuoka, Japan

**Keywords:** green tea, coffee, body iron storage, serum ferritin, iron deficiency

## Abstract

**Purpose:**

This study examined the association between daily green tea and coffee consumption and body iron stores among Japanese middle-aged and older adults.

**Methods:**

This cross-sectional study used data obtained from 2005 to 2007. A total of 10,435 participants were recruited for this study. The participants completed a validated, self-administered food frequency questionnaire on green tea and coffee consumption. A multivariate linear regression analysis was conducted to assess the relationship between green tea and coffee consumption and serum ferritin levels. Additionally, logistic regression analysis was performed to ascertain whether excessive consumption of these beverages was linked to iron deficiency.

**Results:**

We observed that higher green tea and coffee consumption was associated with lower ferritin levels in men and postmenopausal women, even after adjusting for covariates (all *P* for trends <0.05). Among premenopausal women, we found an inverse association between green tea consumption and serum ferritin levels, while no significant association was observed for coffee consumption after adjusting for covariates (green tea, *P* for trend <0.05; coffee, *P* for trend = 0.08). Notably, the association between these beverages and iron deficiency was found only in postmenopausal women; the odds ratios (95% confidence intervals) for iron deficiency associated with almost None, <1 cup/day, 1–2 cups/day, and ≥ 3 cups/day were 1.00 (reference), 0.78 (0.26–2.49), 1.29 (0.49–3.39), and 1.59 (0.63–4.04) (*P* for trend = 0.05), respectively, for green tea and 1.00, 1.32 (0.64–2.73), 1.46 (0.68–3.13), and 2.20 (1.06–4.55) (*P* for trend <0.01), respectively, for coffee.

**Conclusion:**

Higher green tea and coffee consumption was associated with lower serum ferritin levels in men and postmenopausal women. In premenopausal women, consumption of green tea, but not coffee, was associated with lower serum ferritin levels. However, postmenopausal women who ≥3 cups of coffee demonstrated a higher prevalence of iron deficiency compared to those who consumed almost none.

## Introduction

Iron is a critical micronutrient for the sustenance of life (1). However, excessive iron stores in the body have been linked to an increased risk of chronic diseases, such as cardiovascular disease (2–4). Iron plays a vital role in oxygen transport, redox reactions, and nucleic acid synthesis and is particularly instrumental in hematopoiesis (1). Despite its significance, iron deficiency is a prevalent issue worldwide, with research focused on the risk of iron deficiency, particularly in children and women of reproductive ages (5). However, increasing evidence suggests that age-related excessive iron stores are associated with adverse health outcomes, including cardiovascular disease and cancer, due to the overproduction of reactive oxygen species through the Fenton reaction (2–4).

Coffee and green tea are widely consumed beverages and have been suggested to exert protective effects against chronic diseases and mortality (6–10). One possible mechanism for disease risk reduction is that coffee and green tea consumption may reduce oxidative stress by decreasing iron stores in the body by inhibiting iron absorption in the gut (11, 12). Morck et al. (13) first reported in 1983 that consuming coffee with a meat-based meal reduced iron absorption by an average of 39%. Similar results have been reported in several randomized intervention trials in both humans and animals (14, 15). Green tea also inhibits the absorption of non-heme iron; however, the relationship between habitual green tea consumption and body iron stores has been inconsistently reported (14–17). These inconsistent results may be due to insufficient adjustments for confounding factors affecting iron absorption (consumption of coffee and dietary iron intake) or small sample sizes. Furthermore, iron stores in the body differ between men and women, with the iron stores in post-menopausal women approaching those in men (1, 5). Therefore, it is essential to analyze the data stratified by sex and pre- and post-menopausal status. Yet no study has examined these associations while considering the menopausal status.

This study aimed to examine the association between daily coffee and green tea consumption and body iron stores in Japanese individuals stratified by sex and pre- and post-menopausal status. Additionally, we investigated the relationship with iron deficiency, as excessive consumption of these beverages has been reported to lead to iron deficiency.

## Methods

### Study participants

The Japan Multi-Institutional Collaborative Cohort Study, designed as the Saga J-MICC Study, was carried out from2005 to 2007 in the region of Saga, Japan (18, 19). A total of 61,447 registered residents aged 40–69 years were invited by mail to participate in the baseline survey, of whom 12,068 agreed (18). The study’s purpose, content, and conditions were explained in writing and orally to the participants, and they filled out an informed consent form. The research protocol was approved by the Ethics Committee of Saga University Graduate School of Medicine (approval no. 17–11), Nagoya University Graduate School of Medicine (approval no. 253), and the National Institute of Biomedical Innovation, Health, and Nutrition (NIBIOHN-135-01).

Of the 12,068 participants, 1633 were excluded for the following reasons: (i) missing data pertaining to serum ferritin levels (*n* = 55) and habitual coffee and green tea consumption (*n* = 3); (ii) missing data on menopausal status among women (*n* = 9); (iii) any history of a potential inflammatory disease, including cardiovascular disease, cancer, liver disease, or chronic renal failure (*n* = 1,563); and (iv) extremely low or high dietary energy intake (*n* = 3), that is, dietary energy intake less than 800 or equal to 4,000 kcal/day for men and less than 500 or greater than or equal to 3,500 kcal/day for women, as these intake levels are considered impractical and thus potentially questionable in terms of their validity. Finally, 10,435 eligible participants (4,263 men and 6,172 women) were included in the study.

### Data collection

Height and weight were measured to the nearest 0.1 cm, and 0.1 kg, respectively, and body mass index (BMI) was calculated by dividing weight in kilograms by height in meters squared. The participants were also sent a self-administered questionnaire on smoking, dietary habits, current medication, and disease history. Smoking habits were assessed by asking participants about their current smoking status and former smokers’ cessation time. Current and former smokers also reported their usual cigarette consumption level (cigarettes per day) and the age at which they started smoking. Physical activity level (PAL) was assessed using a single-axis accelerometer (Kenz Lifecorder EX; Suzuken Co., Ltd., Nagoya, Japan) on either side of the hip, except when sleeping or bathing, for 10 days after the baseline survey. PAL was calculated by dividing total energy expenditure (kcal/day) by basal metabolic rate (kcal/day). The former was estimated from the accelerometer as the average daily (excluding the first 3 days) energy expenditure, and the latter was defined as basal metabolic standard (20) × body surface area (21) × 24 h.

### Serum ferritin and iron deficiency definition

Venous blood was drawn from each participant, and the serum, plasma, and buffy coat were separated within 3 h and stored at −80°C until testing. A portion of the stored serum specimens was sent to an external laboratory (SRL, Hachioji, Japan), where serum ferritin concentrations were measured using a latex-enhanced immunonephelometric assay on a BN II analyzer (Dade Behring, Marburg, Germany). Iron deficiency was defined as a serum ferritin concentrations <12 μg/L.

### Dietary assessment

The participants’ dietary intake was assessed using a commonly used and previously validated Japanese food frequency questionnaire (FFQ) developed by Tokudome et al. (22, 23). In the FFQ, we asked participants to report their frequency of consumption to assess the average intake of 47 foods and beverages (coffee, green tea, and alcohol) to assess their average intake over the previous year. The FFQ was validated with 3-days weighted diet records as the standard for energy and 26 nutrients, and most of the nutrients showed correlation coefficients of 0.4–0.6 (23). More recently, we conducted to assess the validity of FFQ concerning food group intake, revealing correlation coefficients of 0.17–0.76 for men and 0.23–0.77 for women (24). Notably, the validation of green tea and coffee were relatively high with correlation coefficients, with values of 0.56 and 0.52 for men, and 0.56 and 0.51 for women, respectively (24). The frequencies of coffee and green tea consumption were as follows: almost none, 1–3 times/month, 1–2 times/week, 3–4 times/week, 5–6 times/week, 1 time/day, 2 times/day, and ≥ 3 times/day. Total energy intake was calculated using a program developed at the Department of Public Health, Nagoya City University School of Medicine (22, 23), in accordance with the standard tables of Food Consumption In Japan (fifth revised edition) (20).

### Statistical analyses

All statistical analyses were performed separately according to sex and menopausal status using the SAS statistical software package (ver. 9.4; SAS Institute, Cary, NC, United States). The participants’ coffee and green tea consumption levels were categorized as follows: almost none, <1 cup/day, 1–2 cups/day, and ≥ 3 cups/day. Participant characteristics were compared across coffee and green tea consumption categories using appropriate methods, such as analysis of variance for continuous variables or the Mantel–Haenszel test for categorical variables. The geometric mean values of serum ferritin and their 95% confidence intervals (CIs) were computed for the consumption frequencies of coffee or green tea using the general linear regression procedure of SAS in the three models. The first model controlled for potential confounding factors, including age (years, continuous), BMI (kg/m^2^, continuous), total energy intake (kcal/day, continuous), alcohol status (everyday, sometimes, seldom, or never), smoking status (everyday, sometimes, former, or never), PAL (METs hours/day, continuous), green tea consumption (almost none, <1 cup/day, 1–2 cups/day, or ≥ 3 cups/day), and coffee consumption (almost none, <1 cup/day, 1–2 cups/day, or ≥ 2 cups/day) as covariates in each model. The second model was further adjusted for dietary iron intake (mg/1,000 kcal/day; continuous). In the third model, dietary intake of vitamin C (mg/1,000 kcal/day; continuous) was additionally controlled, as the consumption of dietary vitamin C (ascorbic acid) has an enhancing effect on the absorption of dietary non-heme iron from the gastrointestinal tract (25). To investigate the association between green tea or coffee consumption and iron deficiency, multivariate logistic regression analysis was employed to calculate the odds ratios (ORs) and 95% CIs for iron deficiency related to the consumption frequencies of green tea or coffee. Statistical significance was set at *p* < 0.05.

## Results

The median serum ferritin levels were 115.0 μg/L for men, 13.7 μg/L, and 63.7 μg/L for pre-and postmenopausal women, respectively. Table 1 shows the study participants’ demographic information according to their frequency of green tea consumption. Men who consumed more green tea tended to be older, had a higher total energy, dietary iron, and dietary vitamin C intake, and had lower consumption of coffee; however, they had a lower BMI, and a lower proportion of them were current smokers. A similar trend was observed in both premenopausal and postmenopausal women.

**Table 1:** Characteristics of J-MICC Study Saga participants according to green tea consumption (*n* = 10,435).

Characteristics	Green tea consumption				*P* for trend^*^
	None	<1 cup/day	1**–**2 cups/day	≥3 cups/day	
*Men*, n (%)	450 (10.6)^†^	587 (13.8)	1,432 (33.6)	1794 (42.1)	
Age (years)	54.1 (8.8)	54.5 (8.3)	54.7 (8.1)	57.5 (7.8)	<0.001
Body mass index (kg/m^2^)	23.8 (3.2)	23.8 (3.0)	23.8 (2.9)	23.5 (3.0)	0.009
Current drinker (≥1 *gou*/day)	213 (47.4)	282 (48.0)	661 (46.2)	754 (42.1)	0.054
Current smoker	212 (47.1)	216 (36.8)	519 (36.3)	653(36.4)	<0.01
Total energy intake (kcal/day)	1897 (419)	1879 (343)	1930 (343)	1977 (342)	<0.01
Dietary Fe intake (mg/1,000 kcal/day)	2.91 (0.85)	3.10 (0.72)	3.49 (0.79)	5.11 (1.01)	<0.01
Dietary Vitamin C intake (mg/1,000 kcal/day)	52.7 (16.9)	53.7 (16.2)	62.6 (15.7)	73.6 (17.3)	<0.01
PAL	1.46 (0.11)	1.46 (0.09)	1.46 (0.09)	1.45 (0.09)	0.03
Coffee consumption ≥1 cup/day	267 (59.4)	300 (51.1)	850 (59.4)	886 (49.4)	<0.01
*Premenopausal women*, n (%)	242 (11.4)	323 (15.2)	729 (34.2)	834 (39.2)	
Age (years)	45.0 (3.5)	45.7 (3.9)	46.0 (3.9)	46.6 (4.1)	<0.01
Body mass index (kg/m^2^)	22.1 (3.4)	22.1 (3.3)	22.0 (3.2)	21.8 (3.2)	0.15
Current drinker	25 (10.3)	20 (6.1)	44 (6.0)	47 (5.6)	0.02
Current smoker	48 (19.8)	44 (13.6)	63 (8.7)	78 (9.4)	<0.01
Total energy intake (kcal/day)	1,483 (244)	1,485 (248)	1,512 (232)	1,549 (230)	<0.01
Dietary Fe intake (mg/1,000 kcal/day)	3.68 (0.75)	4.01 (0.81)	4.48 (1.01)	5.35 (1.12)	<0.01
Dietary VC intake (mg/1,000 kcal/day)	3.21 (2.05)	3.29 (1.52)	3.54 (1.75)	3.80 (1.84)	<0.01
PAL	1.47 (0.09)	1.47 (0.08)	1.48 (0.08)	1.47 (0.08)	0.55
Coffee consumption ≥1 cup/day	180 (74.3)	195 (60.4)	544 (74.6)	501 (60.0)	<0.01
*Postmenopausal women*, n (%)	205 (5.1)	405 (10.0)	963 (23.8)	2,471 (61.1)	
Age (years)	57.4 (6.0)	58.8 (5.5)	58.9 (5.6)	60.7 (5.2)	<0.01
Body mass index (kg/m^2^)	22.8 (3.7)	22.9 (3.4)	22.5 (3.0)	22.5 (3.0)	0.03
Current drinker	22 (10.8)	23 (6.0)	39 (4.1)	75 (3.0)	<0.01
Current smoker	38 (18.5)	36 (8.8)	85 (8.8)	128 (5.2)	<0.01
Total energy intake (kcal/day)	1,419 (284)	1,474 (223)	1,496 (232)	1,544 (217)	<0.01
Dietary Fe intake (g/1,000 kcal/day)	4.05 (1.29)	4.15 (1.04)	4.72 (1.13)	5.35 (1.12)	<0.01
Dietary Vitamin C intake (mg/1,000 kcal/day)	57.1 (21.4)	58.6 (19.8)	68.6 (22.0)	81.1 (21.6)	<0.01
PAL	1.47 (0.10)	1.47 (0.08)	1.47 (0.09)	1.46 (0.08)	<0.01
Coffee consumption, ≥1 cup/day	118 (57.6)	224 (55.3)	601 (62.4)	1,164 (47.1)	<0.01

n, number; %, percent; kg, kilogram; kcal, kilocalorie; mg, milligram; PAL, physical activity level.

^*^p-values for linear trends across quartiles (assigned ordinal numbers 0–3) of green tea consumption are based on linear regression analysis for continuous variables and the Mantel test for categorical variables.

^†^Values are the mean (standard deviation) for continuous variables and the number (percentage) for categorical variables.

Table 2 illustrates the geometric mean and 95% CIs of serum ferritin concentrations according to the consumption of green tea or coffee by sex and menopausal status in women. In men, green tea and coffee consumption were significantly negatively associated with serum ferritin levels in the first model (*P* for trend <0.01). Even after adjusting for all factors, these associations remained significant (green tea, *P* for trend = 0.04; coffee, *P* for trend <0.01). Similar associations were observed in postmenopausal women both before (green tea, *P* for trend <0.01; coffee, *P* for trend <0.01) and after (green tea, *P* for trend <0.01; coffee, *P* for trend =0.03) adjustment for potential confounders. In premenopausal women, no association between green tea intake and serum ferritin concentration was observed without adjusting for dietary iron and vitamin C intake; however, after adjustment for dietary iron intake, these associations became significantly inversely associated. These results remained statistically significant with additional dietary vitamin C intake adjustments. In contrast, coffee consumption was not associated with serum ferritin concentration in any model.

**Table 2:** Geometric mean (95% confidence intervals) of serum ferritin levels according to green tea and coffee consumption categories among J-MICC Study Saga participants (*n* = 10,435).

	Habitual consumption				*P* for trend^*^
	None	<1 cup/day	1–2 cups/day	≥3 cups/day	
Men					
Green tea, n	450	587	1,432	1794	
Model 1	106.3 (99.3–115.1)	108.0 (100.9–115.6)	103.3 (98.8–107.9)	99.4 (95.6–103.4)	**0.027**
Model 2	108.2 (99.6–117.6)	109.4 (101.9–117.5)	103.6 (99.1–108.2)	98.3 (94.2–102.6)	**0.015**
Model 3	108.6 (99.8–118.3)	109.7 (102.1–117.9)	103.6 (99.2–108.3)	98.1 (93.8–102.6)	**0.016**
Coffee, n	485	1,475	759	1,544	
Model 1	109.5 (101.4–118.2)	104.9 (100.5–109.6)	104.4 (98.3–110.9)	97.5 (93.4–101.9)	**0.005**
Model 2	109.7 (101.6–118.5)	104.5 (100.6–109.7)	104.5 (98.4–111.0)	97.3 (93.2–101.7)	**0.004**
Model 3	109.7 (101.7–118.5)	105.1 (100.6–109.8)	104.5 (98.4–111.0)	97.3 (93.2–101.6)	**0.004**
Premenopausal women					
Green tea, n	242	323	729	834	
Model 1	12.9 (11.2–14.7)	13.7 (12.2–15.3)	12.9 (12.0–13.9)	12.1 (11.2–13.0)	0.142
Model 2	13.4 (11.7–15.5)	14.1 (12.6–15.9)	13.0 (12.0–14.0)	11.7 (10.9–12.7)	**0.026**
Model 3	13.7 (11.9–15.8)	14.4 (12.8–16.2)	13.0 (12.0–14.0)	11.6 (10.7–12.5)	**0.010**
Coffee, n	131	577	432	988	
Model 1	14.4 (12.1–17.3)	12.8 (11.7–13.9)	13.4 (12.1–14.8)	12.1 (11.4–13.0)	0.102
Model 2	14.7 (12.2–17.6)	12.8 (11.7–13.9)	13.4 (12.1–14.8)	12.1 (11.3–12.9)	0.073
Model 3	14.6 (12.2–17.5)	12.8 (11.7–13.9)	13.4 (12.1–14.8)	12.1 (11.3–12.9)	0.082
Postmenopausal women					
Green tea, n	205	405	963	2,471	
Model 1	66.4 (59.7–74.0)	61.5 (57.1–66.3)	59.0 (56.2–61.9)	57.5 (55.8–59.3)	**0.006**
Model 2	66.8 (61.7–76.8)	63.5 (58.8–68.6)	59.7 (56.8–62.7)	56.8 (55.1–58.6)	**<0.001**
Model 3	68.4 (61.3–76.4)	63.1 (58.4–68.3)	59.5 (56.7–62.5)	57.0 (55.2–58.8)	**<0.001**
Coffee, n	388	1,549	907	1,200	
Model 1	62.5 (57.9–67.5)	58.9 (56.7–61.2)	60.1 (57.2–63.1)	56.3 (53.8–58.8)	**0.036**
Model 2	62.8 (58.2–67.8)	59.0 (56.8–61.3)	60.1 (57.2–63.2)	56.0 (53.6–58.5)	**0.020**
Model 3	62.7 (58.1–67.7)	59.0 (56.8–61.3)	60.1 (57.2–63.2)	56.0 (53.6–58.6)	**0.023**

n, number.

^*^Trend tests were performed by including ordinal numbers 0–3 assigned to each category of habitual consumption in multiple linear regression analysis. Significant *p* values were shown in bold.

Model 1: Adjusted for age (years, continuous), body mass index (kg/m^2^, continuous), total energy intake (kcal, continuous), alcohol consumption (never, former, and current drinkers consuming 0.1–22.9, 23.0–45.9, and ≥ 46 g ethanol/day), smoking (never, former, and current smoker smoking 1–19, 20–39, and ≥ 40 cigarettes per day), physical activity level (continuous), iron intake (mg/1,000 kcal/day), vitamin C intake (mg/1,000 kcal/day), and coffee or green tea consumption (none, <1 cup/day, 1–2 cups/day, and ≥ 3 cups/day).

Model 2: Model 1 plus dietary iron intake (mg/1,000 kcal/day).

Model 3: Model 2 plus vitamin C intake (mg/1,000 kcal/day).

Table 3 shows the multivariate-adjusted ORs and 95% CIs for iron deficiency (ferritin <12 μg/L) according to green tea or coffee consumption. In post-menopausal women, the ORs (95% CIs) for iron deficiency associated with the consumption of none, <1 cup/day, 1–2 cups/day, and ≥ 3 cups/day were 1.00 (reference), 0.78 (0.26–2.49), 1.29 (0.49–3.39), and 1.59 (0.63–4.04) (*P* for trend = 0.05), respectively, for green tea and 1.00, 1.32 (0.64–2.73), 1.46 (0.68–3.13), and 2.20 (1.06–4.55) (*P* for trend <0.01), respectively, for coffee. In contrast, there was no statistically significant association between green tea or coffee consumption and the prevalence of iron deficiency in men and premenopausal women.

**Table 3:** Multivariate-adjusted odds ratios (ORs) and 95% confidence intervals (CIs) of iron deficiency (serum ferritin concentrations <12 μg/L.) according to green tea or coffee consumption in Japanese populations (*n* = 10,435).

		Habitual consumption				
		None	<1 cup/day	1–2 cups/day	≥3 cups/day	*P* for trend^*^
Men	Green tea, n	450	587	1,432	1794	
	No. of iron deficiency (%)	14 (3.1)	13 (2.2)	31 (2.2)	66 (3.7)	
	OR (95% CI)	1.00 (ref)^†^	0.75 (0.34–1.64)	0.73 (0.37–1.44)	1.11 (0.56–2.21)	0.213
	Coffee, n	485	1,475	759	1,544	
	No. of iron deficiency (%)	15 (3.1)	45 (3.1)	18 (2.4)	46 (3.0)	
	OR (95% CI)	1.00 (ref)	1.10 (0.60–2.21)	0.91 (0.44–1.86)	1.22 (0.64–2.30)	0.585
Premenopausal women	Green tea, n	242	323	729	834	
	No. of iron deficiency (%)	111 (45.6)	136 (42.1)	330 (45.3)	404 (48.4)	
	OR (95% CI)	1.00 (ref)	0.84 (0.60–1.19)	0.93 (0.69–1.26)	1.09 (0.81–1.47)	0.192
	Coffee, n	131	577	432	988	
	No. of iron deficiency (%)	55 (42.0)	271 (47.0)	195 (45.0)	460 (46.6)	
	OR (95% CI)	1.00 (ref)	1.28 (0.87–1.91)	1.20 (0.80–1.80)	1.37 (0.94–2.00)	0.185
Postmenopausal women	Green tea, n	205	405	963	2,471	
	No. of iron deficiency (%)	5 (2.4)	8 (2.0)	34 (3.5)	96 (3.9)	
	OR (95% CI)	1.00 (ref)	0.80 (0.26–2.49)	1.30 (0.49–3.40)	1.59 (0.63–4.04)	**0.049**
	Coffee, n	388	1,549	907	1,200	
	No. of iron deficiency (%)	9 (2.3)	46 (3.0)	30 (3.3)	58 (4.8)	
	OR (95% CI)	1.00 (ref)	1.32 (0.64–2.77)	1.46 (0.68–3.13)	**2.20 (1.06–4.56)**	**0.004**

N, number.

^*^Trend tests were performed by including ordinal numbers 0-3 assigned to each category of habitual consumption in multiple linear regression analysis. Significant *p* values were shown in bold.

^†^Adjusted for age (years, continuous), body mass index (kg/m^2^, continuous), total energy intake (kcal, continuous), alcohol consumption (never, former, and current drinkers consuming 0.1–22.9, 23.0–45.9, and ≥ 46 g ethanol/day), smoking (never, former, and current smokers smoking 1–19, 20–39, and ≥ 40 cigarettes per day), physical activity level coffee consumption (none, <1 cup/day, 1-2 cups/day, and ≥ 3 cups/day), iron intake (mg/1,000 kcal/day), and vitamin C intake (mg/1,000 kcal/day).

## Discussion

The present study investigated the relationship between green tea or coffee consumption and the concentrations of serum ferritin by sex and menopausal status in the general healthy population. We found an inverse association between green tea or coffee consumption and serum ferritin levels in men and post-menopausal women. Among premenopausal women, there was a significant negative association between green tea consumption and serum ferritin levels after adjustment for all covariates, including dietary intake of iron and vitamin C. In contrast, post-menopausal women who consumed at least 3 cups of coffee per day had a significantly higher prevalence of iron deficiency compared to those who consumed almost none.

Our findings of an inverse association between coffee consumption and serum ferritin concentration in men and postmenopausal women agree with previous studies (17, 26–28). The prospective cohort study of American adults from the Framingham Heart Study showed a negative association between coffee consumption and serum ferritin level (26). Similarly, Sung et al. reported that increased coffee consumption is associated with decreased ferritin levels (17). Although tea contains high levels of tannins, which are iron absorption inhibitors (29), studies on the relationship between tea intake and serum ferritin levels have shown inconsistent results (30, 31), with some finding a significant association with green tea but not with black or herbal tea. This may be due to differences in the timing and manner of tea consumption and its effect on iron absorption. Green tea is commonly ingested alongside or directly after meals, whereas black tea is typically consumed during intervals between meals. Given that the consumption of these beverages impairs iron absorption in humans when consumed in close proximity to meals (13–15), it is plausible that cultural factor influencing the timing and manner of green and black tea consumption may impact the extent of iron absorption from the diet.

Previous cross-sectional studies have reported that excessive coffee consumption increases the risk of anemia and iron deficiency in populations such as pregnant women (32, 33) and preschool children (34). Our findings indicate that postmenopausal women who consumed ≥3 cups of coffee per day had a significantly higher prevalence of iron deficiency than those who consumed almost no cups of coffee. The regulation of iron metabolism in the body is primarily controlled by hepcidin, and hepcidin increases circulating iron levels by blocking intestinal iron absorption and inhibiting iron release from stores (35). In postmenopausal women, a decrease in estrogen levels has been reported to increase hepcidin production, leading to decreased iron bioavailability (36). Thus, women in the postmenopausal phase may be at increased risk of iron deficiency due to decreased iron absorption from their diet, primarily resulting from excessive coffee consumption, in addition to elevated levels of hepcidin hormones.

*A priori* investigation reported no association between green tea or coffee intake and ferritin levels in a population with relatively severe iron deficiency (31, 37). In the present study, no association was found between green tea consumption and serum ferritin levels in premenopausal women before adjusting for dietary iron intake. There was also a positive association between the number of cups of green tea consumed and iron intake and a significant negative association between green tea consumption and ferritin levels when dietary iron was adjusted for. These findings suggest that dietary iron intake may impact the relationship between green tea consumption and serum ferritin levels in premenopausal women. Furthermore, given that people with low body iron stores and high iron loss rates have increased iron bioavailability compared with those without, dietary iron bioavailability may outweigh the inhibition of iron absorption by green tea and coffee consumption (38, 39).

Our study has several strengths, including a large population sample, the utilization of a validated dietary questionnaire, and an adjustment for potentially important confounding factors. This study also has several limitations. First, the cross-sectional design of our study may have the potential for reverse causation to account for the observed associations. We attempted to minimize the possibility of reverse causation by excluding participants who might have changed their dietary habits as a health-conscious behavior due to having a history of diabetes and hypertension, but the association between green tea and coffee consumption and serum ferritin levels remained statistically significant. Second, green tea and coffee consumption was self-reported; thus, some degree of non-differential misclassification was inevitable. Third, although we adjusted for potential confounding factors in the multivariate analysis, residual confounding factors may have been present due to known or unknown risk factors.

In conclusion, increased coffee and green tea consumption were associated with decreased serum ferritin levels in men and post-menopausal women. In addition, post-menopausal women who drank ≥3 cups of coffee had a higher incidence of iron deficiency than non-coffee drinkers. In premenopausal women, only green tea consumption was negatively associated with serum ferritin levels, and this association may be influenced by dietary iron intake.

## Data availability statement

The original contributions presented in the study are included in the article/supplementary material, further inquiries can be directed to the corresponding author.

## Ethics statement

The study involving human participants were reviewed and approved by the Ethics Committee of Saga University Graduate School of Medicine (approval no. 17-11), Nagoya University Graduate School of Medicine (approval no. 253), and the National Institute of Biomedical Innovation, Health, and Nutrition (NIBIOHN-135-01). The participants provided their written informed consent to participate in this study.

## Author contributions

HN performed the conceptualization, methodology, supervision, writing—original draft, and software validation and formal analysis. HN, MH, YN, YH, and KT performed data collection. HN, MH, CS, and CI performed the data curation. All authors read and approved the final manuscript and contributed to the study’s conception and design.

## Funding

This study was supported by the Grants-in-Aid for Scientific Research for Priority Areas of Cancer (No. 17015018), Innovative Areas (No. 221S0001), and the Japan Society for the Promotion of Science (JSPS) KAKENHI [Nos. 16H06277 (CoBiA) and 20K10547] from the Japanese Ministry of Education, Culture, Sports, Science and Technology. This research was also funded by the Hachiro Honjo Ocha Foundation.

## Conflict of interest

The authors declare that the research was conducted in the absence of any commercial or financial relationships that could be construed as a potential conflict of interest.

## Publisher’s note

All claims expressed in this article are solely those of the authors and do not necessarily represent those of their affiliated organizations, or those of the publisher, the editors and the reviewers. Any product that may be evaluated in this article, or claim that may be made by its manufacturer, is not guaranteed or endorsed by the publisher.
